# Erythrocyte pyruvate kinase deficiency in three West Highland white terriers in Ireland and the UK

**DOI:** 10.1186/2046-0481-66-12

**Published:** 2013-07-10

**Authors:** Florence Juvet, Urs Giger, Ian Battersby, Pierre Menaut, Harriet M Syme, Carmel T Mooney

**Affiliations:** 1 University College Dublin Veterinary Hospital, School of Veterinary Medicine, University College Dublin, Belfield, Dublin, Ireland; 2 Section of Medical Genetics, School of Veterinary Medicine, University of Pennsylvania, Philadelphia, USA; 3 Davies Veterinary Specialists, Manor Farm Business Park, Higham Gobion, Hertfordshire, UK; 4 Department of Veterinary Clinical Sciences, Royal Veterinary College, Hatfield, Hertfordshire, UK

## Abstract

Erythrocyte pyruvate kinase (PK) deficiency is described for the first time in three apparently unrelated West Highland white terriers (WHWT) from Ireland and the UK. All three dogs were diagnosed with markedly regenerative but persistent anaemia and had been treated for presumed immune-mediated haemolytic anaemia (IMHA) before hereditary erythrocyte PK-deficiency was confirmed by breed-specific DNA mutation analysis. This hereditary erythroenzymopathy causes haemolytic anaemia and affects several canine breeds with varying degrees of severity. Although eventually causing osteosclerosis, haemosiderosis and death, PK-deficient dogs can adapt to their anaemia for many years.

PK-deficiency should be considered in anaemic WHWTs worldwide particularly in dogs with haemolytic anaemia where evidence for an immune-mediated, infectious or toxic underlying cause is lacking.

## Background

Hereditary erythroenzymopathies are rare in the general dog population but they may be more common in certain breeds. They must be differentiated from other causes of haemolytic anaemia, including immune-mediated, infectious, toxic and microangiopathic haemolytic anaemias in order to provide appropriate management and advice regarding prognosis and breeding. Mature erythrocytes lack mitochondria and are dependent on anaerobic glycolysis for ATP generation to maintain shape, membrane deformability and active membrane transport [1-3]. Deficiencies in either of the key regulatory glycolytic enzymes, phosphofructokinase (PFK) or pyruvate kinase (PK) result in haemolysis. Such deficiencies are inherited as autosomal recessive traits and are important disorders in several canine breeds [1,4].

The clinical signs, haematological abnormalities and molecular defects of affected dogs have by now been well characterised in several breeds [1]. Specifically, PK-deficiency causes severe but chronic haemolytic anaemia with osteosclerosis and hemosiderosis/-chromatosis. PK-deficiency has been described in Basenjis [5], Beagles [6-8], West Highland white terriers (WHWTs) [9-11], Cairn terriers [12], miniature Poodles [1], Eskimo toys, pugs and Labrador retrievers from the USA [1] and Dachshunds in Germany [1]. The frequency of PK-deficiency in each breed remains unknown as comprehensive surveys have not yet been undertaken to date. This report details the clinical signs and molecular defect of the first identified PK-deficient WHWTs from the UK and Ireland.

## Case presentation

### Case 1

A 1.5-year-old female neutered WHWT was found to have pale mucous membranes during a routine annual examination. The dog was a house pet and its activity level appeared normal according to the owner. Haematological assessment revealed a markedly regenerative anaemia that persisted for one month prior to referral to the University College Dublin Veterinary Hospital, Dublin, Ireland.

The dog was bright and alert, and apart from severe pallor, only hepatosplenomegaly was noted on physical examination. A complete blood cell count (CBC) revealed a haematocrit (Hct) of 0.21 L/L (reference interval (RI) 0.37-0.55 L/L), mean corpuscular volume (MCV) of 96 fL (RI 60–77 fL), mean corpuscular haemoglobin concentration (MCHC) of 275 g/L (RI 320–360 g/L), and an absolute reticulocyte count of 520,000 ×10^6^/L. Microscopic examination of a blood smear revealed normoblastaemia, marked anisocytosis and severe polychromasia with few schistocytes and leptocytes. Other parameters, were within their respective reference intervals. Serum biochemistry depicted only mild hyperbilirubinaemia (16.5 μmol/L; RI 0.9-10 μmol/L), total protein was within reference interval. A direct Coombs’ test was negative. Abdominal ultrasonography and radiography confirmed hepatosplenomegaly but with no other significant abnormalities. In addition, an increased bone opacity was noted in the right femur on the abdominal radiograph. This was further evaluated with radiographs centred on the femur that showed a patchy increased medullary bone opacity. Thoracic radiographs revealed mild right-sided cardiomegaly.

### Case 2

A 2-year-old female WHWT was found to be severely pale during a preoperative assessment for neutering. The anaemia persisted for nine months despite various immunosuppressive treatments (prednisolone 2 mg/kg q24 h for 8 months, azathioprine 2 mg/kg q24 h for 2 months) for presumed immune-mediated haemolytic anaemia (IMHA). The severity of the anaemia (Hct 0.18-0.33 L/L) varied, but the dog continued to be bright and alert. The dog was subsequently referred to Davies Veterinary Specialists, UK to explore other treatment options including splenectomy.

Physical examination revealed pallor, a grade II/VI left sided systolic apical cardiac murmur and palpable hepatosplenomegaly. A CBC revealed a Hct of 0.30 L/L (RI 0.37-0.55), MCV of 80 fL (RI 60–80 fL) and MCHC of 362 g/L (RI 310–370 g/L) (the sample was slightly haemolysed), with marked anisocytosis, polychromasia and rare acanthocytes and schistocytes. There was a stress leucogram, and mild hepatic enzyme elevations were considered likely secondary to previous glucocorticoid therapy. Total protein concentration was within its reference interval. A direct Coombs’ test was negative. Abdominal ultrasonography revealed hepatosplenomegaly but no other significant abnormalities. Thoracic radiographs revealed no abnormalities.

### Case 3

A 2-year-old male entire WHWT was presented to the Queen Mother Hospital, Royal Veterinary College, UK for further evaluation of a persistent regenerative anaemia. The dog had been presumptively treated for IMHA from the age of 10 months (prednisolone 2 mg/kg q24 h for 13 months and cyclosporine 5 mg/kg q24 h for 1 month prior to presentation). Two months previously it had been diagnosed with transient acute pancreatitis (supportive clinical (vomiting and diarrhoea), biochemical and ultrasonographic changes). The dog had also presented one month previously with tachypnoea. Thoracic radiographs revealed a diffuse interstitial pattern and no significant inflammation or evidence of an infectious underlying cause could be determined on BAL.

Physical examination revealed pallor, a grade II/VI left sided systolic apical cardiac murmur and palpable hepatosplenomegaly. A CBC revealed a Hct of 0.29 L/L (RI 0.37-0.55 L/L), a reticulocyte count of 1,421,000 ×10^6^/L, MCV of 65.4 fL (RI 60–77 fL), and MCHC of 329 g/L (RI 310–370 g/L), with marked anisocytosis, polychromasia and few leptocytes and codocytes. Nucleated erythrocytes were also present (8nRBCs/100WBCs). A Coombs‘ test was not performed. A mild neutrophilia was also present (14.7 × 10^9^/L; RI 3–11.5 × 10^9^/L), and serum biochemistry depicted increased serum ALP activity (3,148 U/L; RI 19–285 IU/L), considered likely secondary to previous glucocorticoid administration, and moderate hyperbilirubinaemia (22.2 μmol/L; RI <2.4 μmol/L). Total protein concentration was within its reference interval. Abdominal ultrasonography revealed hepatosplenomegaly but no other significant abnormalities. Thoracic radiographs revealed mild right-sided cardiomegaly and a diffuse interstitial lung pattern. Echocardiography revealed mild eccentric right sided cardiomegaly, thought to be consistent with chronic anaemia. No signs of significant pulmonary hypertension were present.

### Erythrocyte PK mutation analysis

**Figure 1:**
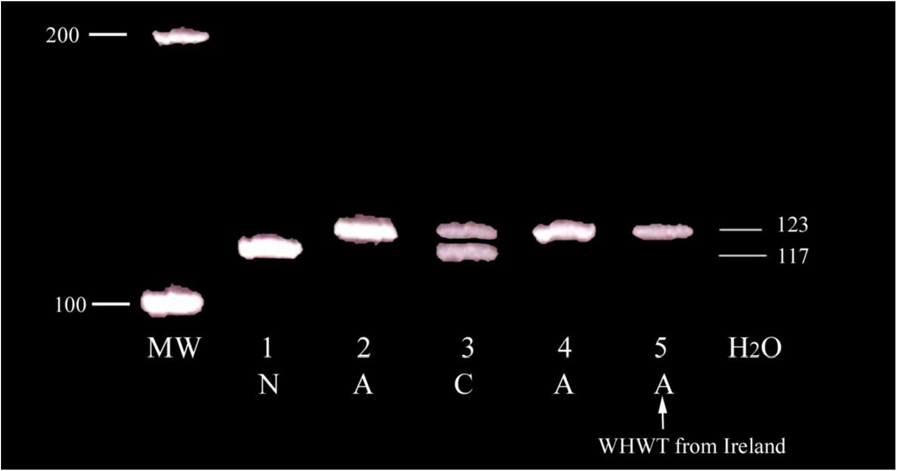
**Results of polyacrylamide gel electrophoresis of genomic DNA amplified by means of the polymerase chain reaction.** N = genotypically normal. A = WHWT affected with PK deficiency. MW + Molecular weight markers, units are base pair (bp). 1 is the negative control, 2 is the positive control, 3 is a carrier (C), 4 is an affected WHWT and 5 is the affected WHWT from Ireland. Note the 6 bp larger band caused by the insertion mutation.

Because of age, breed, negative Coombs’ test results, maintenance of normal demeanour despite persistent anaemia and the absence of response to standard treatment for IMHA, PK- deficiency was suspected in each case. Blood samples collected in ethylenediaminetetraacetic acid (EDTA) were independently submitted from each dog to PennGen laboratories (School of Veterinary Medicine, University of Pennsylvania, Philadelphia) for DNA testing for the mutation previously described in WHWTs in the USA [2,10]. Amplification of genomic DNA from blood yields a single 117 base pair (bp) product for genotypically normal dogs, and a larger product of 123 bp for PK-deficient WHWTs resulting from a 6 bp insertion. Asymptomatic carriers are heterozygous and thus show both products on a gel. The three affected dogs were all found to be homozygous for the 6 bp insertion in the R-PK-gene thereby confirming PK-deficiency (Figure 1).

### Outcome

Pending results of DNA mutation analysis for PK deficiency, all dogs received prednisolone acetate (2 mg/kg PO q24 h), but no other immunosuppressive agents and they remained anaemic. The Hcts oscillated between 0.15 and 0.38 for case 1, 0.19 and 0.33 for case 2 and 0.18 and 0.39 L/L for case 3 over a period of three to 10 months. Prednisolone was discontinued in case 1 and 2 when PK-deficiency was confirmed. Both dogs have remained bright and the owners have reported that quality of life was acceptable with minimal exercise intolerance to date (1 and 3 years later for case 1 and 2, respectively). In case 3, discontinuation of the prednisolone was attempted but was associated with a deterioration of respiratory function and prednisolone was therefore reintroduced at an anti-inflammatory dose (0.625 mg/kg PO q24 h). According to the owners, this dog was well for the next 7 months. Subsequently, respiratory function again declined and the owners elected euthanasia. At that time Hct was 0.28 L/L. Post-mortem examination was declined.

## Discussion

This report details erythrocyte PK-deficiency in three anaemic WHWTs from Ireland and the UK and demonstrates the presence of the same insertion mutation in the R-PK gene as previously reported in WHWTs in the USA [2,10]. As in previous reports, these WHWTs, initially presented with minimal clinical signs or only mild signs of exercise intolerance. The clinical findings of pale mucous membranes and hepatosplenomegaly were similar to previous reports [2,6,8,11,13-15]. Typically, review of the blood smear demonstrates evidence of enhanced erythropoiesis (polychromasia, nucleated RBCs) and echinocytes and other poikilocytes are not seen.

In the WHWT, this disease was first recognised in the USA in 1990 and the causative mutation was identified in 1999 [10]. It is interesting to see that these three European dogs suffered from the same genetic mutation as the previously reported American WHWT. Previous studies have indeed demonstrated that some canine breeds can be separated into different genetic sub-populations corresponding to their geographical origin [16] and that allele frequencies can be different between the US and Europe.

The three cases of this report reportedly had different sires and dams. The Irish dog was known to be from an Irish breeder and both parents were Irish. Because of the diagnosis at different times and in different places it was presumed they were not closely related but extended pedigrees were not available. To date, other family members have not been made available for DNA testing, although the breeders were contacted and common ancestry is possible. However, the diagnosis of PK-deficiency in these three cases suggests that it should be considered in any WHWT with persistent regenerative anaemia for which an underlying cause is unknown. Currently breeders in the UK and Ireland are not generally screening their WHWTs, although this case series and another recent report outside the USA [11] would suggest value in doing so worldwide. Erythrocyte PK-deficiency in WHWTs occurs as an autosomal recessive trait. A DNA test performed on 100–200 microlitres of EDTA blood or cheek swabs enables differentiation of affected (homozygous), carrier (heterozygous) and healthy (homozygous normal) dogs. Several laboratories currently offer the test and an up-to-date list of available DNA tests for hereditary disease in dogs can be found at http://research.vet.upenn.edu/WSAVA-LabSearch.

Because the mutations are different from breed to breed [2,11], the activity and stability of the PK enzyme is more or less affected. As a consequence the disease manifests with slightly varying degrees of severity between affected populations. In humans, over 125 mutations have been identified causing variable disease severity [17]. In affected canine breeds only one mutation per breed has been reported to date. In some closely related breeds (e.g. Cairn terriers and WHWTs) the mutation is identical [2]. Despite the variation in severity, the condition is eventually fatal as worsening anaemia and/or hepatic failure secondary to hemosiderosis/-chromatosis [1,2] are seen. Interestingly, in contrast to other species [18,19], PK-deficient dogs have not been reported to develop gall bladder calculi.

The three cases presented here demonstrate that in WHWTs, PK-deficiency may be detected incidentally upon discovering pallor despite no or minimal clinical signs. PK-deficient dogs can adapt well to the anaemia as their haemoglobin readily releases oxygen because of a high 2,3-diphosphoglyceride content and right shifted haemoglobin-oxygen dissociation curve [20]. If life-threatening crises can be avoided (avoiding the demands of strenuous exercise and preventing and controlling other concurrent diseases rapidly) a reasonable longevity can be achieved despite persistent anaemia. Indeed, life-expectancy can be longer in WHWTs compared to other affected canine breeds with the longest surviving WHWT reaching 9 years of age [2]. Thus the prognosis can be more predictable than for IMHA, which often has a more acute onset, severe anaemia and guarded prognosis. Indeed, two of the affected cases in this report remained alive and well for several years and the remaining case that was euthanased was for a reason unrelated to the anaemia.

In the three cases described herein, prolonged treatment with prednisolone and/or azathioprine may have been responsible for some of the concurrent abnormalities detected (e.g. hepatomegaly, pancreatitis). However, most changes were likely because of extramedullary erythropoiesis and hemosiderosis/-chromatosis (hepatosplenomegaly) and chronic anaemia (mild right sided cardiomegaly, cardiac murmur). Not unexpectedly, concurrent and unrelated diseases are also possible in these cases. One dog suffered from tachypnoea, which was thought to be secondary to a respiratory disease. A definitive diagnosis was not made but pulmonary fibrosis was suspected given the breed, radiographic findings and exclusion of inflammatory or infectious causes.

There is no specific, only supportive therapy for PK deficiency, but blood transfusions are rarely required as the dogs appear to adapt well to their chronic anaemia. Chronic glucocorticoid therapy may inhibit macrophage function and thus premature removal of PK-deficient erythrocytes, but can be associated with undesirable complications and, as illustrated in this report, can be safely discontinued providing there are no other therapeutic indications for their use. Other immuno- and bone marrow-suppressive drugs are potentially harmful in PK-deficient dogs as they can suppress the bone marrow response, may induce additional adverse effects and can significantly increase unnecessary expense. Splenectomy has not been shown to be effective in slowing the degree of haemolysis in PK-deficient dogs [20] compared to humans and cats. Iron chelation may be considered when iron overload is excessive, but affected dogs appear to be fairly resistant to the development of hemochromatosis, probably because of a shorter iron plasma clearance time than in healthy dogs [20]. In humans iron overload is most common after repeated transfusions [2,12]. Experimental bone marrow transplant can be curative [21,22], however the difficulty in finding a compatible donor, the intensive therapy required for bone marrow ablation and continued immunosuppression, the high mortality rate, and extreme costs make it an unrealistic option for most owners. Recently, PK-deficient dogs have been used in gene therapy experiments, and results were promising [23].

The development of osteosclerosis as noted in one case reported here and myelofibrosis is a unique feature observed only in PK-deficient dogs [2], but not PK-deficient cats and humans^1^. Its pathogenesis is unclear.

Recognition of erythrocyte PK-deficiency amongst other causes of haemolytic anaemia is critical. The finding of anaemia in an otherwise reasonably healthy young WHWT as illustrated in this report should arouse suspicion for the disorder. In PK-deficient dogs, a Coombs’ test and a search for infectious diseases and toxicity will be negative providing evidence of an alternative diagnosis to other causes of haemolytic anaemia.

## Conclusions

This reports demonstrates the occurrence of PK deficiency in WHWTs native to the UK and Ireland. A single DNA test performed at any age even before signs occur can confirm the diagnosis thereby saving costly investigations and avoiding inappropriate potentially harmful and expensive treatments. Early recognition and testing of parents and siblings should help prevent propagation of the disease in the breed by identifying affected and carrier animals and eliminating them from breeding programmes or mating carriers with homozygous healthy individuals and testing all their offspring prior to continued breeding.

## Competing interests

The authors declare that they have no competing interests.

## Authors’ contributions

FJ was the lead author. IB, PM and HMS provided additional case details. UG carried out the molecular genetic studies. CTM and UG supervised production of the manuscript. All authors participated in drafting the manuscript. All authors read and approved the final manuscript.
